# Supplementary material for: Inflammation as a Sex-Specific Mediator in the Relationship between Maternal and Offspring Obesity in C57Bl/6J Mice
Source: Biology (Basel). 2024 May 31;13(6):399. doi: 10.3390/biology13060399 (PMC11200566; doi:10.3390/biology13060399)
Supplement: Supplementary file 1 [file biology-13-00399-s001.zip › biology-3029551-supplementary.pdf]

Supplemental Table 1. RT-qPCR Gene Targets

Gene Name	Gene Symbol	Assay ID	Catalog #
Interleukin 1 beta	Il1b	Mm00434228_m1	4331182
Interleukin 6	Il6	Mm00446190_m1	4331182
Tumor Necrosis Factor	Tnf	Mm00443258_m1	4331182
18S ribosomal RNA	Rn18s	Mm03928990_g1	4331182

TaqMan gene expression assays were purchased from ThermoFisher Scientific, Carlsbad, CA.
